# Neem Leaf (*Azadirachta indica* A. Juss) Ethanolic Extract on the Liver and Kidney Function of Rats

**DOI:** 10.1155/2021/7970424

**Published:** 2021-03-30

**Authors:** Irma Seriana, Muslim Akmal, Darusman Darusman, Sri Wahyuni, Khairan Khairan, Sugito Sugito

**Affiliations:** ^1^Graduate School of Mathematics and Applied Sciences, Universitas Syiah Kuala, Banda Aceh 23111, Indonesia; ^2^Department of Midwifery, Polytechnic of Health, Ministry of Health, Aceh Besar 23231, Indonesia; ^3^Laboratory of Histology, Faculty of Veterinary Medicine, Universitas Syiah Kuala, Banda Aceh 23111, Indonesia; ^4^Soil Science Department, Faculty of Agriculture, Universitas Syiah Kuala, Banda Aceh 23111, Indonesia; ^5^Laboratory of Anatomy, Faculty of Veterinary Medicine, Universitas Syiah Kuala, Banda Aceh 23111, Indonesia; ^6^Department of Pharmacy, Faculty of Mathematics and Natural Sciences, Universitas Syiah Kuala, Banda Aceh 23111, Indonesia; ^7^Herbal Medicinal Research Centre, Universitas Syiah Kuala, Banda Aceh 23111, Indonesia; ^8^Atsiri Research Centre, Universitas Syiah Kuala, Banda Aceh 23111, Indonesia; ^9^Laboratory of Pathology, Faculty of Veterinary Medicine, Universitas Syiah Kuala, Banda Aceh 23111, Indonesia

## Abstract

Neem (*Azadirachta indica* A. Juss) is one of the tropical plants found in Indonesia that has been used to prevent and treat various diseases. This study aimed to investigate the effect of the ethanol extract of neem leaves on the concentration of aspartate aminotransferase (AST), alanine aminotransferase (ALT), urea, and creatinine in male rats. Twenty-four male Wistar rats were randomly divided into four groups (T0, T1, T2, and T3) with 6 rats in each group. T0 is the control group, and T1, T2, and T3 are the treatment groups that were administered 100, 200, and 300 mg/kg body weight of neem leaf ethanolic extracts for 48 days, respectively. On day 49, blood samples were collected to measure the concentration of AST, ALT, creatinine, and urea followed by an evaluation of liver and kidney histology. The results showed that the ethanolic extract of neem leaves did not affect the concentration of AST, ALT, and creatinine, The ethanol leaves reduced extract on the urea concentration, no abnormal changes were observed in the liver and kidney organs. In the future, it is required to carry out a comprehensive safety evaluation of the neem leaf ethanol extract for herbal medicines.

## 1. Introduction

The development of medicines from natural products has the world's attention today. The natural products have a unique chemical structure, produce a diversity of biological activities, and have drug-like properties [1]. The practice of medicine using natural products occurs since prehistoric times in preventing and treating various diseases. Natural products offer many advantages in terms of efficiency and selectivity of the molecule target so that they can be used repeatedly to supply the urgent need of medicines effectively [2]. Natural products contain active compounds with various pharmacological activities against various diseases without or with minimal side effects [3].

Neem (*Azadirachta indica* A. Juss) is native to India, the family Meliaceae is widespread in the world, and it can grow in most tropical and subtropical countries, including Indonesia. In Indonesia, neem is known locally as Imba, Nimba, or Mimba [4]. Neem has great medical benefits through its various biological properties [5]. Almost all parts of the neem plant can be used to treat various diseases [6]. Based on the previous reports, the neem leaves extract have numerous biological and pharmacological activities including antipyretic, analgesic, antihepatotoxic [7], spermicide, anti-implantation [8, 9], antihyperglycemic, antiulcer, antifungal, antibacterial, anti-inflammatory, immunomodulatory, antimutagenic, anticancer, antimalarial, antiviral, antioxidant [10–12], antifertility [13, 14], and contraception [9, 11].

Various parts of the neem plant have been successfully isolated, contain more than 140 chemical compounds [10], and have been used as herbal medicines for thousands of years [14]. Neem contains various primary compounds including fat derivatives, carbohydrates, and proteins and secondary compounds such as flavonoids, steroids, saponins, terpenoids, alkaloids, glycosides, and tannins [7–15]. Neem plants are a worldwide interest because of their efficacy without showing side effects. The use of the neem plant traditionally is quite safe, and more than 75% of traditional medicine uses the neem leaf extract. The history of its use, from existing reports, does not indicate any side effects from neem leaves. However, comprehensive safety evaluation of the use of neem leaf formulations has never been done [11].

The previous study reports that the neem leaf extract has toxic effects on the liver and kidney [4, 11]; however, other reports show that the neem leaf extract can protect the liver and kidney from damage [16–18]. In Aceh Besar Regency, Aceh Province, Indonesia, the local people have used neem leaves as vegetables and used to prevent pregnancy without knowing the side effects. The safety of neem leaves to date is still unclear. Until now, to our knowledge, there are still limited studies that evaluate the effect of the neem leaf ethanolic extract on the liver and kidney function. In the current study, the objective was to investigate the effect of administration of the neem leaf ethanolic extract on the liver and renal function through examining the concentration of AST, ALT, urea, and creatinine and histopathological liver and kidney in male rats.

## 2. Materials and Methods

### 2.1. Neem Leaf Sample Collection

The neem leaves were collected from Kajhu Village, Aceh Besar Regency, Aceh Province, Indonesia, in September 2019. Identification of neem leaves was carried out at Bogoriense Herbarium, Biology Research Center, Indonesian Institute of Sciences (LIPI), Bogor, Indonesia (1454/IPH.1.01/If.07/VII/2019), and it is indicated as *Azadirachta indica* A. Juss.

### 2.2. Preparation of the Ethanol Extract of the Neem Leaf

Fresh neem leaves were collected, washed, air-dried, and mashed to produce a crude powder. Furthermore, crude powder was macerated with ethanol 70% for 7 days. Then, the filtrate was evaporated by using a rotary evaporator at 50°C to produce viscous extracts.

### 2.3. Animal Experiment

The experimental animals were 24 male Wistar rats with 150–250 g body weight. Rats were acclimatized for 7 days and randomly divided into four groups (T0, T1, T2, and T3) as each group contained 6 rats. T0 is the control group without treatment, and T1, T2, and T3 are the treatment groups that were administered the neem leaf ethanolic extract with 100, 200, and 300 mg/kg body weight, respectively. The extract was administered orally using a gastric tube for 48 days, and blood samples were taken on the 49th day to determine the concentrations of AST, ALT, urea, and creatinine, and then liver and kidney tissues were collected to evaluate histopathology. During the study, rats were given standard feed and water ad libitum.

### 2.4. Biochemical Analysis

The blood samples (2–3 ml) were taken from the heart using a sterile syringe. Then, blood was transferred to the Eppendorf tube and left at room temperature (30°C) for 10 minutes. Blood was centrifuged for 10 minutes at 3000 rpm. The serum was separated and stored in a refrigerator (−20°C). Furthermore, AST, ALT, urea, and blood creatinine were examined using a chemistry analyzer (Rayto RT-1904C). The determination procedure of AST, ALT, urea, and creatinine concentrations was performed according to the manufacturer's protocol.

### 2.5. Histopathology Examination

The liver and kidney of rats were fixed in 10% neutral buffered formalin solution for 24 h, dehydrated in an alcohol series, and cleared in xylol solution followed by embedding on the paraffin block. Tissues embedded in the paraffin block were cut using a rotary microtome into 4 *µ*m thick, mounted on glass slides, stained with hematoxylin and eosin, and examined under a light microscope at a magnification of 10 × 10.

### 2.6. Statistical Analysis

The data are analyzed using analysis of variance (one-way ANOVA) followed by Duncan's post hoc test using SPSS 24. A *p* value <0.05 was considered statistically significant.

## 3. Results and Discussion

### 3.1. The  AST  and  ALT  Concentration

The AST  concentration of T0, T1, T2, and T3 was 290.9 ± 202.5 U/L, 277.2 ± 167.2 U/L, 421.3 ± 201.5 U/L, and 366.3 ± 276.9 U/L, respectively. The concentration of AST in T2 and T3 groups tended to increase compared to T0 (control group). However, AST concentrations in T1, T2, and T3 groups showed no significant difference from the T0 group (*p* > 0.05). The ALT concentration of T0, T1, T2, and T3 was 108.6 ± 23.1 U/L, 116.3 ± 51.9 U/L, 105.9 ± 15.2 U/L, and 95.9 ± 36.2 U/L, respectively. The concentration of ALT in T2 and T3 groups tended to decrease compared to T0 (control group). However, ALT concentrations in T1, T2, and T3 groups showed no significant difference from the T0 group (*p* > 0.05) (Table 1).

The AST enzyme is one marker that was used to assess the presence of liver damage. This enzyme played a role in the process of gluconeogenesis to catalyze the conversion of the aspartic acid amino group into ketoglutaric acid to produce oxaloacetic acid. AST enzyme is found in cytosolic and mitochondrial isoenzymes of the liver, skeletal muscles, heart muscles, kidneys, brain, pancreas, lungs, leukocytes, and red blood cells. However, this AST enzyme is less sensitive and specific for assessing liver damage [19].

Although there were no significant differences in AST concentrations in the treatment groups (T1, T2, and T3) compared to the control group (T0), the AST concentration tended to increase in the T2 and T3 groups compared to T0 (control group). The increase was possibly late due to the active compounds of the neem leaf extracts such as flavonoids, tannins, saponins, alkaloids, and steroids, but the active compounds of neem leaves ethanol extract is not identified to increase AST. An increase in the AST enzyme is not necessarily followed by liver damage because this enzyme is not only specific in the liver but also in other body tissues.

This study also found the ALT enzyme tended to decrease in the treatment group (T1, T2, and T3) compared to the control group (T0), but the decrease in the ALT enzyme was not statistically significant. ALT enzymes were a better predictor of liver damage than AST enzymes because this cytosolic enzyme was found in the highest concentrations in the liver and was more specific in assessing the damage of liver function [20]. This enzyme played a role in gluconeogenesis by catalyzing the transfer of amino groups from alanine to ketoglutaric acid to produce pyruvic acid [19]. ALT consisted of 496 amino acids and was found in the cytosol hepatocytes. ALT enzyme activity in the liver was about 3000 times of serum activity, so in the case of hepatocellular damage or death, ALT released from damaged liver cells will increase the measured activity of the ALT enzyme in the serum [21].

Mohamed et al. [22] found that the ethanol extract of the neem leaf caused a decrease in rat AST and ALT enzymes. The ethanol extract of the neem leaf did not have side effects on the liver, and it even serves as liver protection. The study conducted by Haque et al. [23] also found that the neem leaf extract did not cause changes in the normal serum activity of AST and ALT enzymes in male rats. Bhanwra et al. [24] reported that administering 500 mg/kg body weight of the neem leaf extract in paracetamol-induced rats decreased liver damage, as indicated by normal AST and ALT concentrations and histopathological observations. The results of the study conducted by Dkhil et al. [17] indicated a protective effect of the methanolic extract of neem leaves with 500 mg/kg body weight dose to the liver on the rat induced by ciplastin. Mallick et al. [25] reported that the methanolic extract of the neem leaf had no toxic effect on the rat liver even at high doses. The antioxidant content of the neem leaf extract gave a hepatoprotective effect.

The study conducted by Hartono and Prabowo [26] found that the administered ethanol extract of the neem leaf on rat by induced high doses of paracetamol for ten days could also decrease the activity of the enzyme ALT in experimental animals. The decrease in the ALT enzyme was due to the active compound of the neem leaf extract, which functioned as hepatoprotection by inhibiting oxidative liver damage. The antioxidants could reduce free radical formation by direct scavenging which is reducing the formation of reactive oxygen species (ROS) which have toxic effects on the membrane phospholipids and cause a broad spectrum of cell damage. The antioxidants can reduce oxidative stress to the liver cells, and the activity of the ALT enzyme in blood will decrease.

### 3.2. The Urea and Creatinine Concentration

**Figure 1:**
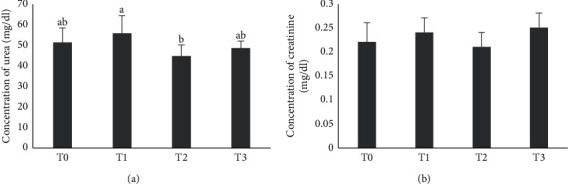
The concentration (mean ± SD) (*n* = 6) of urea (a) and creatinine (b) after administration of neem leaf ethanolic extracts in male rats. Different superscripts indicate a statistically significant difference between groups (*p* < 0.05). T0 = control group, T1 = 100 mg/kg treated group, T2 = 200 mg/kg treated group, and T3 = 300 mg/kg treated group.

The urea concentration of T0, T1, T2, and T3 was 51.1 ± 7.0 mg/dl, 55.6 ± 8.5 mg/dl, 44.5 ± 5.4 mg/dl, and 48.4 ± 3.4 mg/dl, respectively. Urea concentrations in T1, T2, and T3 groups showed significant difference from the T0 group (*p* < 0.05). The creatinine concentration of T0, T1, T2, and T3 was 0.22 ± 0.04 mg/dl, 0.24 ± 0.03 mg/dl, 0.21 ± 0.03 mg/dl, and 0.25 ± 0.03 mg/dl, respectively. Creatinine concentrations in T1, T2, and T3 groups showed no significant difference from the T0 group (*p* > 0.05) (Figure 1).

The kidney has an important role in the excretion of waste products and toxins such as urea, creatinine, and uric acid; it also has a function in the regulation of extracellular fluid volume, osmolality, electrolyte concentrations, and hormone production. The functional unit of the kidney is the nephron consisting of the glomerulus, proximal tubules, distal tubules, and collecting ducts [27]. The results showed that there were significant differences in urea concentrations in the treatment groups (T1, T2, and T3) compared to the control group (T0), and the urea concentration decreased in the T2 and T3 groups compared to T0 (control group). Urea is a nitrogen-containing compound formed in the liver as the final product of protein metabolism and the urea cycle. About 85% of the urea is excreted through the kidneys, and the rest is excreted through the digestive tract. Serum urea increases in kidney cleansing conditions as in acute and chronic kidney failure. Urea can also increase in other conditions such as upper gastrointestinal bleeding, dehydration, catabolic process, and high-protein diet [27].

In this study, there was a decrease in the concentration of urea in the treatment group compared to the control group. This indicated that the ethanol extract of the neem leaf did not affect the renal function of the male rat. According to Guyton and Hall [28], total urea in blood was determined by dietary protein and the ability of the kidneys to excrete urea. If the kidneys were damaged, urea would accumulate in blood. Increased urea in plasma indicated kidney failure in carrying out its filtration function. The condition of kidney failure, which is characterized by very high plasma urea levels, is known as uremia.

Urea is the final product of nitrogen derived from protein and amino acid catabolism, produced by the liver, and distributed to all intracellular and extracellular fluids. In the kidney, urea is filtered out of blood by glomeruli and partly reabsorbed by water. Increased urea is an indicator that the glomerular filtration rate is poor. Increased urea concentration is associated with kidney disease or failure, urinary tract obstruction by kidney stones, congestive heart failure, dehydration, fever, shock, and bleeding in the digestive tract.

The study also found that there were no significant differences in creatinine concentrations in the treatment groups (T1, T2, and T3) compared to the control group (T0). This indicated that the ethanol extract of neem leaves did not cause damage to the rat kidney. Creatinine is commonly used as a measure of kidney function. Creatinine concentration is not only influenced by the product of muscle mass but also influenced by muscle function, muscle composition, activity, diet, and health status. Increased creatinine concentrations are also found in muscular dystrophy, anemia, leukemia, and hyperthyroidism [29]. Creatinine is the most commonly used marker for assessing renal function. The creatinine concentration provides an indicator of the action of the glomerulus. Creatinine is a byproduct of creatine phosphate in muscles and is produced at a constant speed by the body. For the most part, creatinine is cleared from blood entirely by the kidneys. Increased creatinine shows decreased cleansing by the kidneys. Creatinine serum is a more accurate assessment of kidney function than urea [27].

Moneim et al. [16] found that administering the neem leaf extract at a dose of 500 mg/kg body weight for five days in rats did not cause changes in urea and creatinine concentrations in the rat. Kpela et al. [30] also found that the rat fed with the neem leaf extract at 500 mg/kg body weight dose for 14 days caused a decrease in urea and creatinine concentrations in the rat induced by cisplatin for kidney damage. The ethanol extract of the neem leaf acted as nephroprotection in kidney damage. The study conducted by Somsak et al. [18] found that the neem leaf water extract at doses of 1000 and 2000 mg/kg body weight did not have toxic effects on rats. The neem leaf extract can be used as a candidate for nephroprotection against damage to the kidneys.

### 3.3. The Liver and Kidney Histopathology

**Figure 2:**
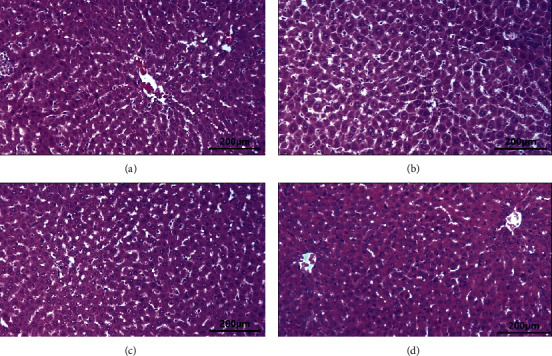
The liver histology after administration of neem leaf ethanolic extracts in male rats: (a) control group, (b) treated group = 100 mg/kg, (c) treated group = 200 mg/kg, and (d) treated group = 300 mg/kg.

**Figure 3:**
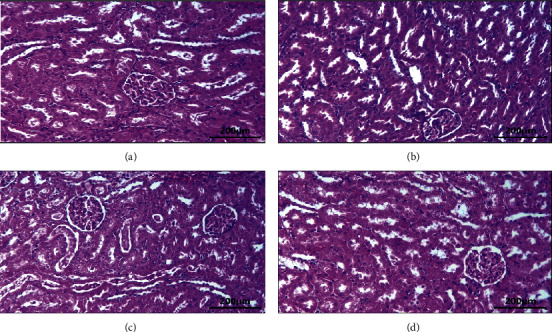
The kidney histology after administration of neem leaf ethanolic extracts in male rats: (a) control group, (b) treated group = 100 mg/kg, (c) treated group = 200 mg/kg, and (d) treated group = 300 mg/kg.

The liver histopathological observations in the control group (Figure 2(a)) and the treatment group at doses of 100 mg/kg (Figure 2(b)), 200 mg/kg (Figure 2(c)), and 300 mg/kg (Figure 2(d)) showed that the liver, central veins, and hepatocyte cells appeared normal (Figure 2). The kidney histopathology observations in the control group (Figure 3(a)) and the treatment group at doses of 100 mg/kg (Figure 3(b)), 200 mg/kg (Figure 3(c)), and 300 mg/kg (Figure 3(d)) showed normal appearance of the glomerulus and proximal and distal tubules. There was no histological change in the kidney (Figure 3).

The liver and kidneys are organs that have an important role in the metabolism and excretion of drugs or other substances. In the study, it was shown that the administration of the ethanol extract of neem leaves at doses of 100, 200, and 300 mg/kg did not cause abnormalities and damage to the rat liver. The liver in treated rats showed the normal general structure of the liver and normal appearance of central veins and hepatocyte cells. The liver histopathology is following the results of the analysis of the AST and ALT enzymes which showed that there was no significant difference between the control and treatment groups. Although there was an increase in the AST enzyme in the 200 and 300 mg/kg group compared to the control group, the liver histopathology appeared normal. AST  enzyme is not specific for liver damage. AST is found primarily in the red blood cells, cardiac and skeletal muscles, and kidneys. AST is less sensitive to the liver than ALT [19].

The kidney histopathology observations after administration of the ethanol extract of neem leaves with doses of 100, 200, and 300 mg/kg showed no difference compared to the control. The kidney in treated rats showed the normal general structure of the kidney and normal appearance of the glomerulus and tubules. This finding is supported by the analysis of urea and creatinine in rat blood which showed no significant difference between the control and treatment groups. Urea and creatinine are biochemical parameters that are main indicators of kidney function. However, creatinine is a more accurate assessment of kidney function than urea [27]. This is in line with some of the previous reports that the neem leaf extract has potential hepatoprotective and nephroprotective effects [31]. Kusuma et al. [32] found that the neem leaf ethanol extract did not affect hepatocyte diameter, liver lobules' diameter, and liver weight in the rat. In contrast, the study of Katsayal et al. [33] found that the administration of the methanol extract of neem leaves at doses of 500, 1000, and 2000 mg/kg caused abnormalities in the liver and kidneys of treated rats compared to controls. This difference in results was probably due to the different extract dosages of the neem leaves.

The results of this study indicate that the ethanol extract of neem leaves from Kajhu did not cause damage to the liver and kidneys of male rats. This is due to the active compound of the ethanol extract of neem leaves which is an antioxidant. The previous studies show that the neem leaf ethanol extract from Kajhu contains flavonoids, alkaloids, tannins, saponins, and steroids [34]. The active compounds that act as antioxidants are flavonoids. The neem leaf ethanol extract has strong antioxidant activity [35]. Thus, the ethanol extract of neem leaves from Kajhu is safe for the liver and kidneys of male rats.

## 4. Conclusions

The ethanolic extract of neem leaves did not cause damage to the liver and kidney of the male rat as indicated by the analysis of AST, ALT enzymes, urea, and creatinine concentration and histopathological liver and kidney. However, further investigation is required for comprehensive safety evaluation of the neem leaf extract at various doses to obtain a clear picture of the safety of neem leaves for medicine.

## Figures and Tables

**Table 1:** The concentration of AST and ALT in the male rats after administering the neem leaf ethanol extract.

Parameters	T0	T1	T2	T3
AST (U/L)	290.9 ± 202.5	277.2 ± 167.2	421.3 ± 201.5	366.3 ± 276.9
ALT (U/L)	108.6 ± 23.1	116.3 ± 51.9	105.9 ± 15.2	95.9 ± 36.2

Data are presented as mean ± SD (*n* = 6). There was no significant difference between groups (*p* > 0.05). T0 = control group; T1 = 100 mg/kg treated group; T2 = 200 mg/kg treated group; T3 = 300 mg/kg treated group.

## Data Availability

The data used to support the findings of this study are available from the corresponding author upon request.
